# Effect of the p53–tristetraprolin–stathmin-1 pathway on trophoblasts at maternal–fetal interface

**DOI:** 10.1371/journal.pone.0179852

**Published:** 2017-06-28

**Authors:** Xiao-Ling Ma, Xiao-Cui Li, Fu-Ju Tian, Si-Ming Zhang, Xiao-Rui Liu, Yan Zhang, Jian-Xia Fan, Yi Lin

**Affiliations:** 1 Institute of Embryo-Fetal Original Adult Disease, The International Peace Maternity & Child Health Hospital, Shanghai Jiao Tong University School of Medicine, Shanghai, China; 2 Shanghai First Maternity and Infant Hospital, Tongji University School of Medicine, Shanghai, China; 3 Department of Obstetrics and Gynecology, Renmin Hospital of Wuhan University, Wuhan, China; Hokkaido Daigaku, JAPAN

## Abstract

**Problem:**

To reveal the effect of p53–tristetraprolin–stathmin-1 signaling on trophoblasts and recurrent spontaneous abortion (RSA).

**Method of study:**

Stathmin-1 (STMN1), p53, and tristetraprolin (TTP) expression in paraffin-embedded villus tissue was determined using immunohistochemistry. HTR-8/SVneo cells were treated with doxorubicin to activate p53; STMN1 and TTP levels were detected by quantitative reverse transcription–PCR and western blotting. Western blotting and immunofluorescence were used to investigate STMN1 expression after TTP overexpression or knockdown in HTR-8 cells.

**Results:**

STMN1 was downregulated and p53 was upregulated in the villus tissue from patients with RSA. Doxorubicin decreased STMN1 expression but enhanced TTP expression in HTR-8 cells. *In vitro*, TTP overexpression inhibited STMN1 production; TTP knockdown promoted it. TTP downregulated STMN1 expression in trophoblasts by directly binding its 3ʹ untranslated region.

**Conclusions:**

TTP modulates trophoblast function and interacts with STMN1 and p53, and is related to pregnancy outcomes.

## 1 Introduction

Miscarriage, the most common complication of pregnancy, is defined as the spontaneous loss of pregnancy before the fetus has reached viability [1]. Recurrent spontaneous abortion (RSA) is defined as the occurrence of two or more consecutive pregnancy losses before 24 weeks of gestation [2]. In recent years, many molecules (including eukaryotic translation initiation factor 5A [eIF5A] [3] and peroxiredoxin 2 [4]) and immune cells such as dendritic cells, natural killer (NK) cells [5, 6], invariant NK T (iNKT) cells [7,8], T helper (Th)1, and Th17 cells [9] have been linked with RSA.

Stathmin-1 (STMN1), also referred to as OP18, is a ubiquitous cytosolic phosphoprotein, proposed to be a small regulatory protein and a relay integrating diverse intracellular signaling pathways involved in controlling cell proliferation, differentiation, and activities [10]. In rodent uteri, STMN1 expression is upregulated at the embryo implantation site and may play a role in the early stages of pregnancy [11]. Recent reports have shown differential STMN1 expression in human endometrial and placental cells and that it may participate in decidualization. We found that STMN1 was downregulated in the chorionic villi from patients with RSA. It is a trophoblast proliferation- and invasion-associated microtubule regulatory protein that participates in the pathogenesis of RSA [12].

Many studies have suggested that STMN1 is a target of p53. Alli and colleagues found that silencing STMN1 induced a tumor suppressor function in breast cancer cell lines harboring mutant p53 (p53^MUT^) [13]. Another study showed that p53 inhibits STMN1 expression and that it is associated with G2/M block [14]. STMN1 is also believed to be important in ovarian cancer by increasing p53^MUT^ protein stability and transcriptional activity [15]. The p53 gene, first described in 1979, was the first tumor suppressor gene identified [16]. The p53 protein regulates the course of transcription of many genes in response to a wide variety of stress signals. Following DNA damage, p53 regulates key processes, including DNA repair, cell cycle arrest, senescence, and apoptosis, thereby suppressing cancer [17].

p53 is a potential mediator of pregnancy via estrogen and progesterone activation [18]. Studies on the p53 signaling pathway have suggested an association between polymorphisms and infertility, implantation failure after *in vitro* fertilization, and RSA. It is believed that p53 controls the apoptosis pathways (p53–cyclin-dependent kinase inhibitor [CDKN]1A and p53–Bax) and that it may be involved in the pathogenesis of RSA [19,20].

The regulation of mRNA turnover is critical for controlling gene expression. The 3′ untranslated regions (3′ UTRs) of the mRNAs of transiently expressed genes frequently contain an AU-rich sequence. The AU-rich elements (AREs) are the recognition signal for an mRNA processing pathway that specifically degrades the mRNAs of certain lymphokines and cytokines, including the T cell–specific cytokine interleukin (IL)-2 [21]. A set of RNA-binding proteins termed AU-binding proteins (AUBPs) specifically bind to AREs, either stabilizing mRNAs or promoting their destruction [22].

Different signaling pathways may participate in mammalian cell mRNA decay, including ARE-mediated mRNA decay [23]. Tristetraprolin (TTP/Zfp36) is a phosphorylating protein that recruits one or more degradative enzymes and initiates mRNA decay. TTP binds to ARE-containing mRNAs, marking them for delivery to processing bodies (P-bodies), where transcripts are degraded by mRNA decay enzymes [24]. TTP mRNA and protein levels are increased in the reproductive tract of mice during the estrous cycle [25]. In addition, the TTP family member TIS11D is crucial to female fertility and embryonic development, as disrupted TIS11D resulted in infertility in mice [26].

Previously, we found that STMN1 plays a key role in regulating trophoblast invasion and that impaired STMN1 expression may lead to RSA [12]. Little is known about the effects of p53 and STMN1 on trophoblast migration during pregnancy. We aimed to reveal the effects of p53–TTP–STMN1 signaling on trophoblast function and pregnancy outcomes.

## 2 Materials and method

### 2.1 Cell culture

The HTR-8/SVneo cells (HTR-8, human extravillous trophoblast cell line) were a generous gift from PK Lala (University of Western Ontario, Ontario, Canada). The cells were cultured in phenol red Dulbecco’s modified Eagle’s medium (DMEM)/F12 supplemented with 10% fetal bovine serum (FBS, Gibco, Grand Island, NY, USA), streptomycin (10 μg/mL), and penicillin (100 U/mL) at 37°C with 5% CO_2_.

The cells were seeded onto 6-well plates and incubated overnight at 37°C with 5% CO_2_. The cells reached approximately 60–70% confluence after 24 h. Then, the cells were changed to new DMEM/F12 supplemented with 10% FBS and 0.1, 0.25, 0.5, 1.0, 1.5, or 2.5 μmol/mL doxorubicin (Sigma-Aldrich, St. Louis, MO, USA). The cells were harvested at 6 h, 12 h, and 24 h after treatment. mRNA was analyzed by quantitative real-time PCR (qRT-PCR); proteins were analyzed by western blotting and immunofluorescence.

### 2.2 Human sample characteristics

We recruited 18 patients with RSA, age 23–37 years, who had been treated at the Department of Obstetrics and Gynecology of the International Peace Maternity & Child Health Hospital of the China Welfare Institute, Shanghai Jiao Tong University School of Medicine, China, between February 2015 and May 2016. Medical examination excluded classic risk factors such as abnormal parental karyotypes, uterine anatomical abnormalities, infectious diseases, luteal phase defects, diabetes mellitus, thyroid dysfunction, and hyperprolactinemia.

We also recruited 18 women aged 22–34 years with normal early pregnancies as the healthy controls. These women had all had previous pregnancies without any history of RSA, preterm labor, or pre-eclampsia. Induced abortion was performed by suction apparatus to terminate their unwanted pregnancy at 8–12 weeks of gestation, and samples of villous tissue were collected and stored in liquid nitrogen.

The Medical Ethics Committee of International Peace Maternity & Child Health Hospital of the China Welfare Institute, Shanghai Jiao Tong University School of Medicine approved this study. Written informed consent was obtained from all patients who participated in the study.

### 2.3 qRT-PCR

Total RNA was extracted from the cultured cells using TRIzol (Life Technologies, Grand Island, NY, USA) according to the standard method and complementary DNA (cDNA) was generated using a PrimeScript II 1st Strand cDNA synthesis kit (Takara, Dalian, China) using random or oligo-dT primers. qRT-PCR was performed using a SYBR Green kit (Takara). The forward and reverse primers used were as follows: *STMN1*: 5′-AGCTGGCTGAGAAACGAGAG-3′ and 5′-AGTCTCGTCAGCAGGGTCTT-3′; *TTP*: 5′-CGAAGGGCCACTCCTATCAG-3′ and 5′-CCGCTGCTGGCATATTCATC-3′; glyceraldehyde-3-phosphate dehydrogenase (*GAPDH*): 5′-TGGAGTCCACTGGCGTCTTC-3′ and 5′-TGCTGATGATCTTGAGGCTGTTG-3′. For the *in vitro* experiments, the relative expression level was calculated using the comparative threshold cycle (2^-ΔΔCt^) method and normalized to the internal control gene *GAPDH* (human).

### 2.4 Immunohistochemistry

Chorionic tissues were collected and subsequently fixed, embedded, sectioned, deparaffinized, rehydrated, and unmasked, using standard immunohistochemical techniques, using a BB-SA-1052 detection kit (Boster, Wuhan, China) according to the manufacturer’s instructions. The primary antibodies used were rabbit anti-STMN1 (1:250; Abcam, Cambridge, UK), anti-p53 (1:160; Cell Signaling Technology, Beverly, MA, USA), and anti-TTP (1:200; Santa Cruz Biotechnology, Dallas, TX, USA).

### 2.5 TTP and p53 overexpression or knockdown

Lentiviruses carrying short hairpin RNA (shRNA) and overexpressing lentiviral vectors targeting human TTP were purchased from GeneChem (Shanghai, China) and transduced into the HTR-8 cells according to the manufacturer’s instructions. Control siRNA and on-target individual siRNAs against p53 were synthesized by Shanghai GenePharma (Shanghai, China) and transfected into the cells at a final concentration of 100 nmol/L using Oligofectamine reagent (Life Technologies).

### 2.6 Immunofluorescence

HTR-8 cells were cultured on poly-L-lysine–coated coverslips in 24-well plates, treated with control vector (vector) or lentiviruses carrying short hairpin RNA (shTTP), cultured for 48 h, washed three times with phosphate-buffered saline (PBS), fixed in 4% paraformaldehyde–PBS for 10 minutes, and stained with primary antibodies [rabbit anti-STMN1 monoclonal antibody (1:150; Abcam) and mouse anti-TTP monoclonal antibody (1:100; Santa Cruz Biotechnology)] overnight at 4°C. The next day, secondary fluorescent Alexa Fluor 488 donkey anti-rabbit IgG (H+L) and Alexa Fluor 594 donkey anti-mouse IgG (H+L) antibodies (Life Technologies) were used after the plates had been washed three times with PBS. Nuclei were counterstained using 4,6-diamidino-2-phenylindole (DAPI; Abcam). Coverslips were mounted on the glass slides and the slides were visualized using a Leica microscope (Wetzlar, Germany). For the chorionic tissues, rabbit anti-p53 monoclonal antibody (1:1600; Cell Signaling Technology) was used as primary antibody.

### 2.7 Western blotting

Total protein from HTR-8 cells was prepared using 1× sodium dodecyl sulfate (SDS) lysis buffer (60 mmol/L Tris-HCl [pH 6.8], 10% glycerol, 2% SDS, sodium salt, 0.01% bromophenol blue, 100 mmol/L DL-dithiothreitol). Then, the mixture was heated in a water bath for 8 min at 100°C. Equal amounts of protein samples were subjected to 12% polyacrylamide gel electrophoresis and transferred onto polyvinylidene difluoride (PVDF) membranes (Bio-Rad Laboratories, Richmond, CA, USA). Then, 5% non-fat milk in Tris-buffered saline with Tween (TBST) was used to block the PVDF membranes for 1 h at room temperature. The membranes were incubated with antibodies against STMN1 (1:2000; Abcam), TTP (1:500; Abcam), p53 (1:1000; Cell Signaling Technology), p-p53 (1:1000; Cell Signaling Technology), p21 (1:1000; Cell Signaling Technology), α-tubulin (1:1000; Yeasen, Shanghai, China), or GAPDH (1:1000; Cell Signaling Technology) overnight at 4°C, followed by horseradish peroxidase (HRP)-conjugated secondary antibody (1:2000; Yeasen) for 1 h. Signals were detected using a Prolight HRP chemiluminescent kit (Tiangen Biotech, Beijing, China) according to the manufacturer’s instructions [27].

### 2.8 Luciferase assay

To generate the STMN1 3′ UTR–luciferase reporter constructs, a ~1.5-kb DNA fragment containing TTP binding sites was amplified and cloned into a pGL3 luciferase vector (Promega, Madison, WI, USA). STMN1 3’ UTR constructs carrying mutations in the putative TTP binding sites were generated using a QuikChange Lightning Multi Site-Directed mutagenesis kit (Stratagene, La Jolla, CA, USA). HTR-8 cells were transfected with the luciferase reporter plasmids and the vector or TTP overexpression plasmids, and were harvested after 24 h. Luciferase activity was measured using a dual luciferase reporter assay kit (Promega).

### 2.9 Statistical analyses

Statistical analysis was performed using GraphPad Prism version 6.0 (GraphPad, La Jolla, CA, USA). Experiments were performed in technical duplicates of at least three biological replicates. All results are reported as the mean ± SD [28]. All *P*-values are two-sided. Student’s *t*-test was used to determine significant differences. *P* < 0.05 was considered significant.

## 3 Results

### 3.1 STMN1 is downregulated and p53 is upregulated in patients with RSA

**Fig 1:**
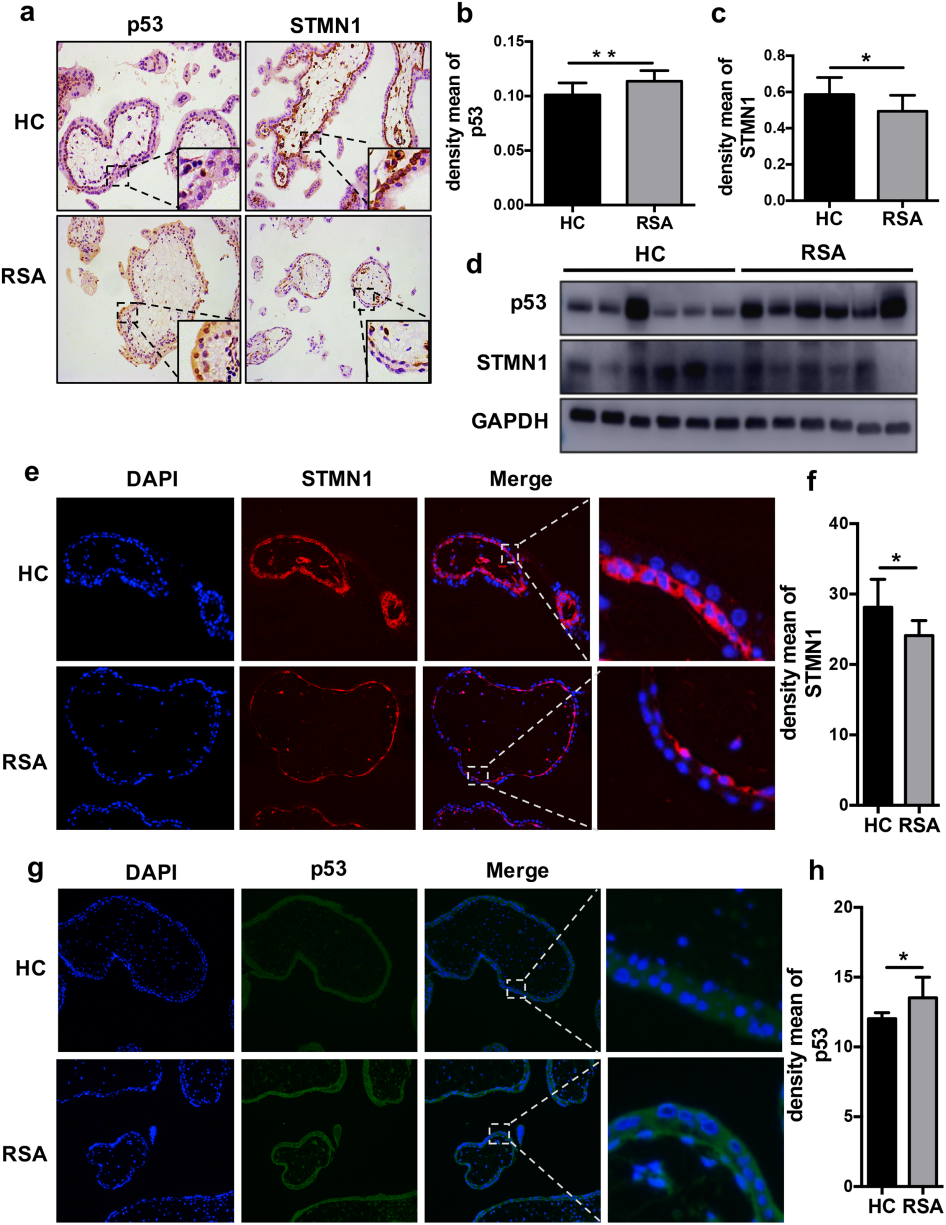
STMN1 is downregulated and p53 is upregulated in patients with RSA. (a-c) Immunohistochemistry (*n* = 12), (d) western blotting (*n* = 6), and (e-g) immunofluorescence analyses of paraffin-embedded villus tissue showed that in RSA, increased p53 expression (green) and decreased STMN1 expression (red) in RSA as compared with the healthy controls (HC) (*n* = 8). (b, h) Density mean of p53 staining was quantified with Image-Pro Plus 6.0. (c, f) Density mean of STMN1 staining was quantified with Image-Pro Plus 6.0. Original magnification: ×20 (a, e, g). *P < 0.05; **P < 0.01.

We investigated the STMN1 and p53 expression patterns at the maternal–fetal interface in patients with RSA. Immunohistochemistry and immunofluorescence analysis showed that STMN1 levels were significantly decreased in the patients with RSA as compared to the healthy controls (Fig 1a, 1c, 1e and 1f). On the contrary, p53 levels were increased in the patients with RSA (Fig 1a, 1b, 1g and 1h). The western blotting studies confirmed these findings. p53 was upregulated and STMN1 was downregulated in the villus tissue from patients with RSA (Fig 1d).

### 3.2 Doxorubicin activation of p53 reduces STMN1 expression in trophoblasts

**Fig 2:**
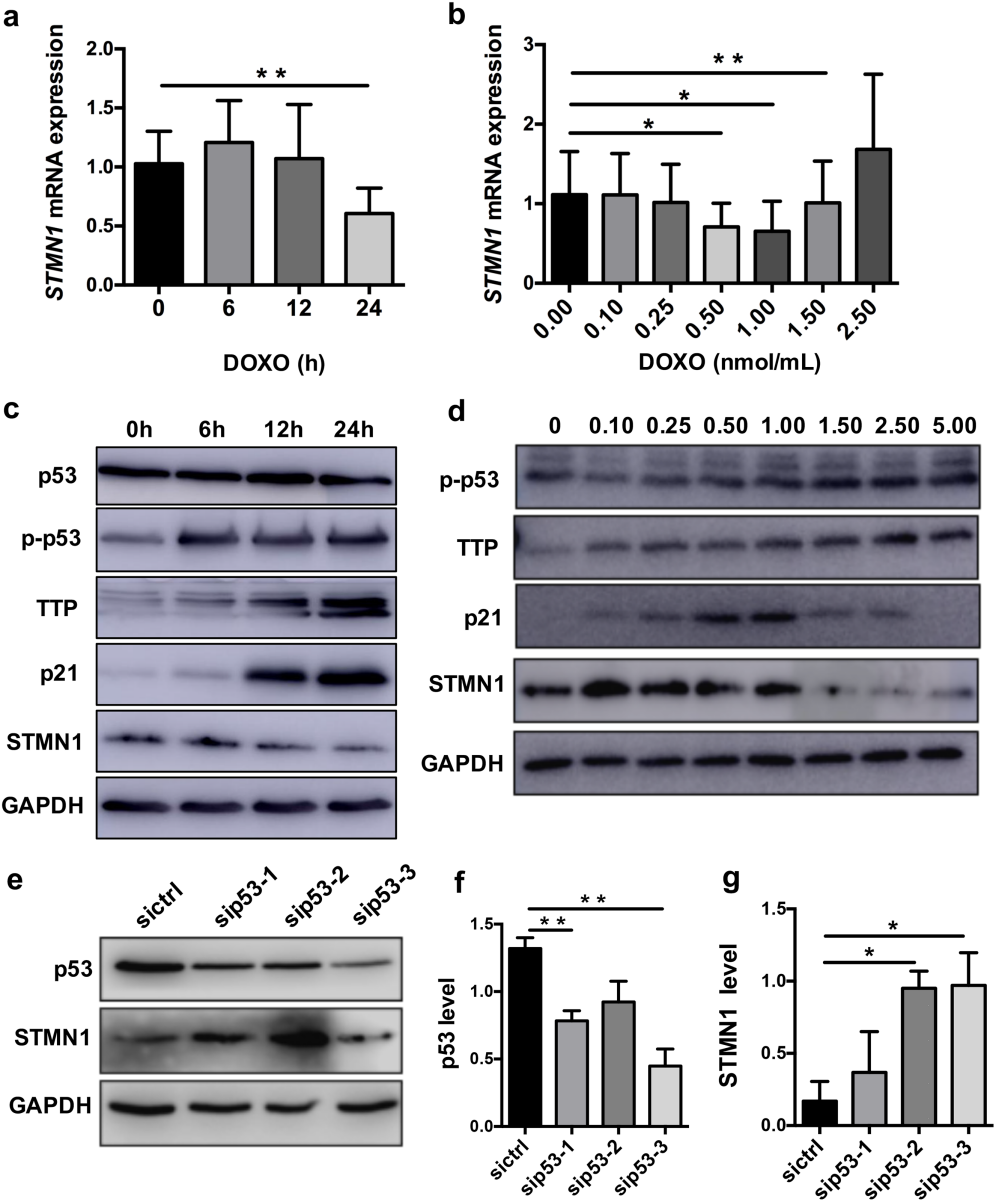
Doxorubicin (DOXO) decreases STMN1 and increases TTP in HTR-8 cells. (a, b) qRT-PCR and (c, d) western blotting showed the tendency effects of timing at 1.0nmol/mL and doxorubicin concentration at 24h. (e-g) p53 knockdown increased STMN1 expression in HTR-8 cells. (f-g) Histogram showing the relative expression level of p53 and STMN1 protein in HTR-8 cell as determined using Image J. The data represent the means ± SD of five (a, b) and three (f, g) independent experiments. *P < 0.05; **P < 0.01 versus control cells.

Doxorubicin intercalates into DNA and inhibits the production of topoisomerase II (an enzyme that unwinds DNA), which halts DNA replication and induces the accumulation and transcriptional activity of p53 [29]. To determine the relationship between p53 and STMN1, we used the HTR-8 cell line, a first trimester human extravillous cytotrophoblast-derived cell line, to construct a cell model in which doxorubicin activates p53 *in vitro*. First, we added doxorubicin to the HTR-8 medium and harvested the cells at 6 h, 12 h, and 24 h to perform qRT-PCR and western blotting. qRT-PCR showed that STMN1 expression was obviously decreased at the optimal concentration of 0.5–1.5 μmol/mL doxorubicin and at the optimal time point of 24 h (Fig 2a and 2b). The western blotting results conformed to that of the qRT-PCR and doxorubicin treatment had no impact to expression of p53 but increased the expression of phosphor p53 (p-p53) and p21 (Fig 2c and 2d). Transfection with p53 siRNA (sip53) or control siRNA (sictrl) revealed that STMN1 was upregulated after knockdown of the p53 gene (Fig 2e–2g). The results suggest that excessive p53 production may inhibit STMN1 in trophoblasts.

### 3.3 TTP is upregulated in patients with RSA and activates p53 via doxorubicin *in vitro*

**Fig 3:**
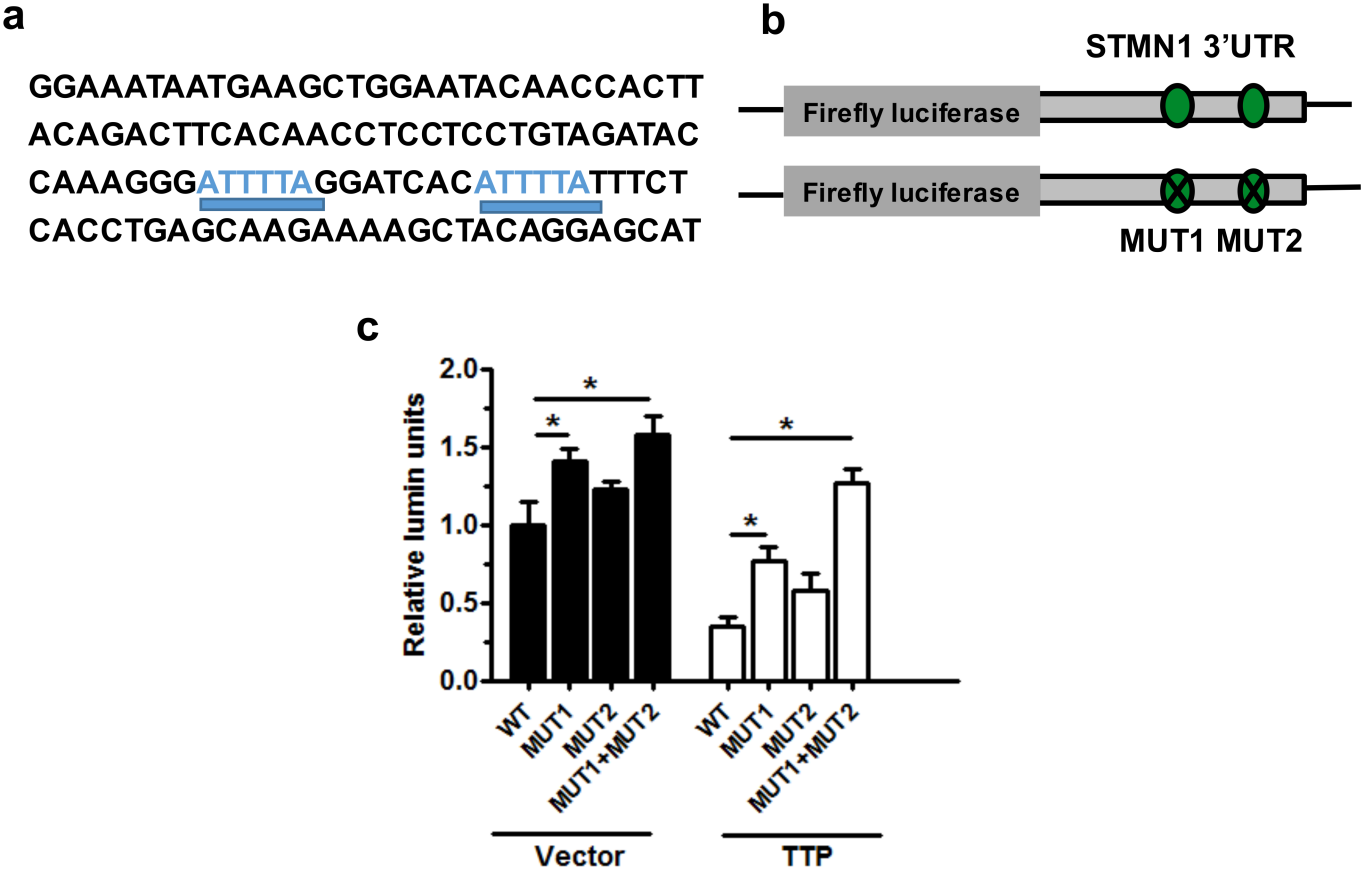
STMN1 was an authentic target of TTP. (a) Two predicted TTP binding sites within the human *STMN1* 3'UTR (underlined in blue). (b) Construction of the wild-type *STMN1* 3'UTR reporter (WT) and the TTP binding site–deleted mutant reporters (MUT1 and MUT2). (c) Luciferase assay. TTP overexpression dramatically altered STMN1 expression. The data represent the means ± SD of three independent experiments. *P < 0.05 versus the vector control.

**Fig 4:**
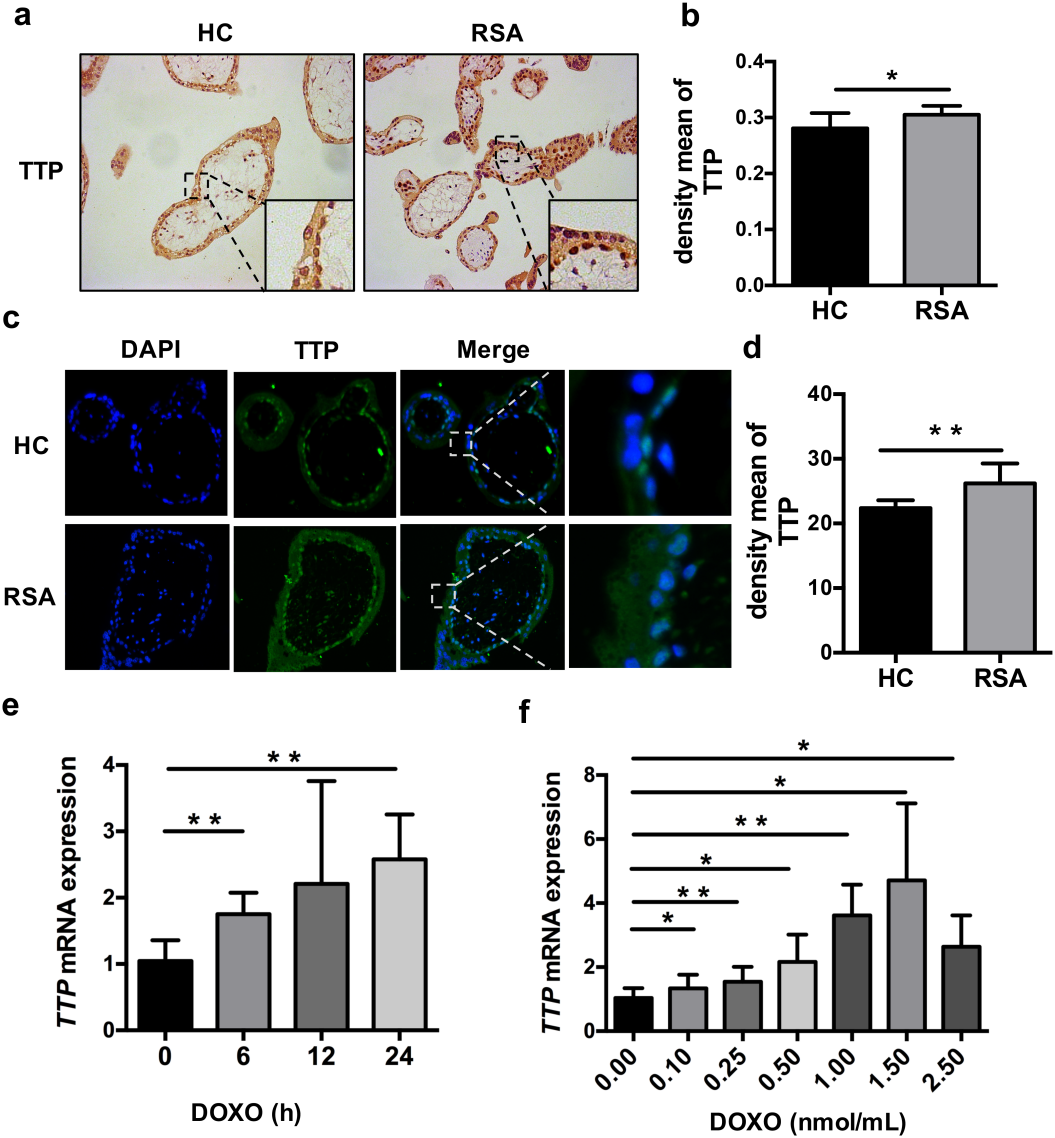
TTP expression patterns in patients with RSA and doxorubicin (DOXO) upregulation of TTP expression in HTR-8 cells. (a, b) Immunohistochemistry (*n* = 12) and (c, d) immunofluorescence assays (*n* = 8) of paraffin-embedded villus tissue revealed higher TTP expression (green) in patients with RSA than in the healthy controls (HC). (e, f) qRT-PCR showing that TTP was increased in response to doxorubicin treatment in a time- and concentration-dependent manner. The data represent the means ± SD of five independent experiments. (b, d) Density mean of TTP staining was quantified with Image-Pro Plus 6.0. Original magnification: ×20 (a, c). *P < 0.05; **P < 0.01.

To investigate the relationship between p53 and STMN1, we searched the literature and found a key molecule: TTP. Prediction of the human STMN1 region using bioinformatics software *TRANSFAC* (Transcription factor database) showed that STMN1 3’UTR region has two TTP binding sites (Fig 3a). It may be the key of linking p53 with STMN1. To confirm our hypothesis, we subjected paraffin-embedded tissue to immunohistochemistry and immunofluorescence assays. TTP levels were significantly higher in the patients with RSA than in the healthy controls (Fig 4a–4d). qRT-PCR and western blotting were performed to explore TTP expression after doxorubicin treatment in HTR-8 cells. TTP was significantly increased in a time- and concentration-dependent manner (Figs 2c, 2d, 4e and 4f). Interestingly, the change in TTP expression was the opposite of that of STMN1 and was in keep with p53 levels. As previously reported, TTP is an ARE-mediated regulator of mRNA decay [23]. Our findings indicate that TTP regulates STMN1 and that STMN1 expression in trophoblasts correlates inversely with TTP levels in patients with RSA.

### 3.4 TTP regulates STMN1 expression *in vitro*

**Fig 5:**
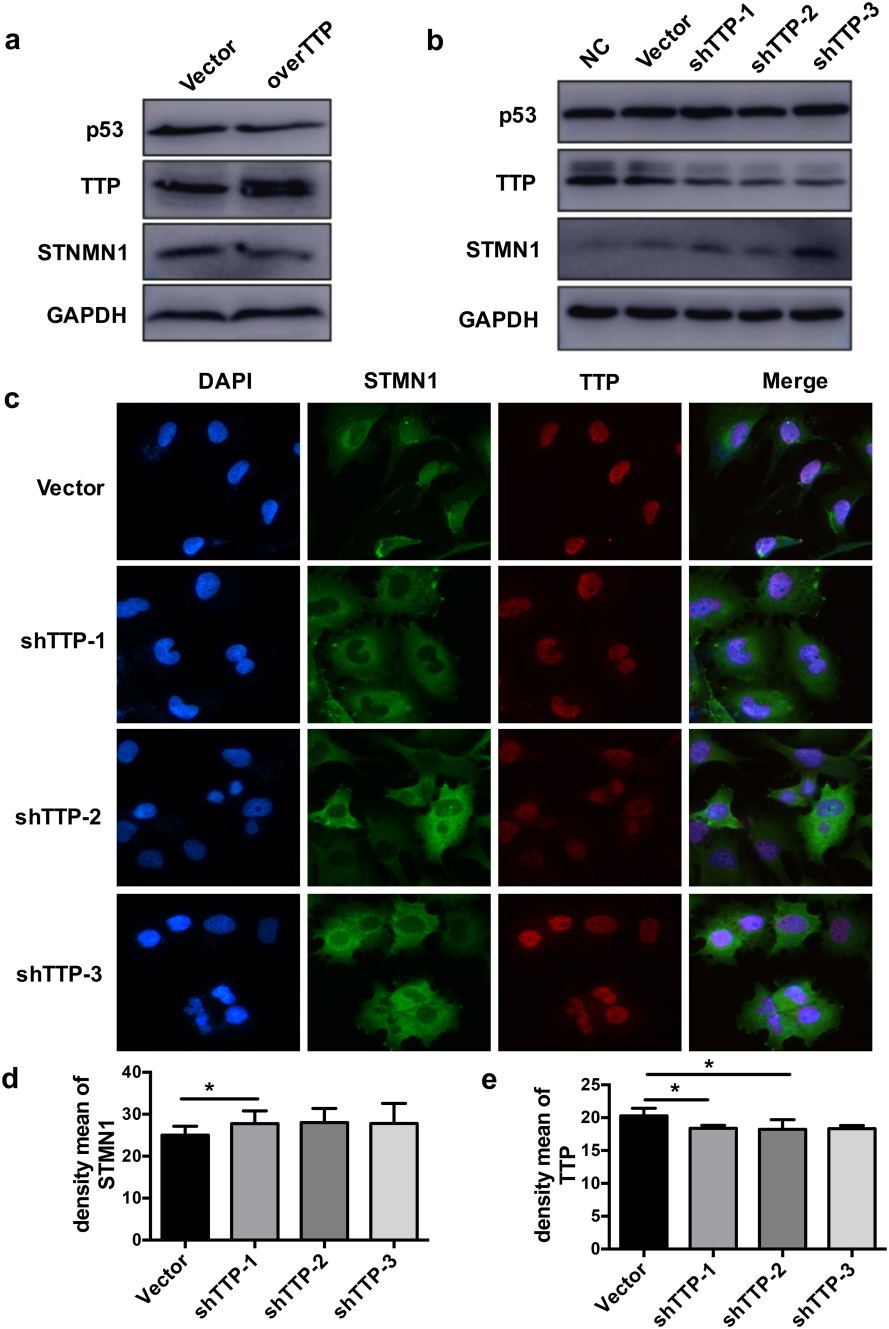
TTP regulates STMN1 expression *in vitro*. (a) TTP overexpression (overTTP) reduced STMN1 expression, but did not affect p53 expression. (b) TTP knockdown (shTTP) increased STMN1 expression, but did not affect p53 expression. (c-e) Bicolor immunofluorescence staining after TTP knockdown, cells were subjected to for STMN1 (red), TTP (green) and counterstained with DAPI (blue). (d, e) Density mean of STMN1 and TTP staining were quantified with Image-Pro Plus 6.0. The data represent the means ± SD of three independent experiments. Original magnification: ×20 (c). *P < 0.05.

To gain further insight into the relationship between TTP and STMN1, HTR-8 cells were transduced with shTTP or TTP-overexpressing lentiviral vector, and incubated for 48–72 h. TTP expression was upregulated after transfection of the TTP-overexpressing vector and was decreased after shTTP transduction (Fig 5a, 5b, 5c and 5e). Western blotting showed that TTP overexpression in HTR-8 cells resulted in decreased STMN1 expression as compared with the control cells, but did not affect p53 expression (Fig 5a). Western blotting and bicolor immunofluorescence assay showed that TTP knockdown in HTR-8 cells increased STMN1 expression as compared with the control cells, but did not affect p53 expression (Fig 5b–5e). Obviously, TTP did not directly regulate p53 expression. Furthermore, three luciferase reporters were constructed with a control of either the wild-type human *STMN1* 3'UTR (WT reporter) or two mutants in which two putative TTP binding sites had been deleted (MUT1 and MUT2 reporter, respectively) (Fig 3a and 3b). As expected, STMN1 expression was increased in HTR-8 cells which were transfected with MUT1 or MUT2 reporter as compared with transfection in WT reporter. Moreover, the simultaneous addition of MUT1 and MUT2 had an obvious effect on STMN1. In a rescue experiment using TTP overexpression, MUT1, MUT2, and MUT1+MUT2 transfection significantly decreased STMN1 expression as compared with transfection in the vector control cells (Fig 3c). Our findings strongly imply that STMN1 is an authentic target of TTP and that TTP may regulate trophoblast function and may be involved in the pathogenesis of RSA.

## 4 Discussion

Successful embryo implantation depends on embryo hatching, trophoblast development, and proper maternal–fetal cross-talk and immune regulation. The invasive potential of trophoblasts is essential for embryo implantation [30]. In our previous study, gain- and loss-of-function analyses showed that STMN1 regulates trophoblast proliferation and migration *in vitro*. The present study demonstrates that TTP affects the changes in STMN1 expression at the maternal–fetal interface in patients with RSA.

TTP is now known to bind to so-called class II AREs within the mRNAs that encode tumor necrosis factor (TNF)-α and granulocyte/macrophage colony–stimulating factor (GM-CSF). TTP protein is localized in both the nuclear and cytosolic compartments in fibroblasts, and its translocation from the nucleus to the cytosol can be accomplished rapidly, i.e., within 5 min, in response to stimuli such as polypeptide growth factors and phorbol esters, but not by agents that elevate cyclic adenosine monophosphate (cAMP) levels [31]. Cytokines are a prime target of TTP. A well-characterized example is TNF-α, whose expression is efficiently controlled by TTP as a part of a negative feedback loop [32]. TTP modulates various other inflammatory mediators, such as macrophage inflammatory protein-2 (MIP-2) [33], GM-CSF [34], interferon-γ [35], IL-22 [36], IL-10 [37], IL-6 [38], and post-transcriptional gene regulation is frequently organized in functional units. *TTP* is believed to play a key role in tumorigenesis, as its mRNA encodes the LATS2 tumor suppressor and the E6-AP ligase that directs p53 destruction [39]. Interestingly, a recent report on the TTP family and their selected targets during porcine pregnancy found that the shift in TTP expression and TIS11D points to a potential role of these genes in regulating spontaneous fetal loss [40]. However, little is known about its function in human trophoblasts at the maternal–fetal interface.

p53 is required for TTP induction in cancer cells [41], and doxorubicin enhances p53 activity [14], suggesting that doxorubicin may induce TTP expression in trophoblasts. Accordingly, we designed *in vitro* cell culture experiments to verify this premise. Our results showed that, in HTR-8 cells, doxorubicin increased both mRNA and protein levels of TTP in a dose- and time-dependent manner. Previously, we had found that STMN1 expression was significantly decreased in uterine NK cells from CBA/J × DBA/2J mouse matings as compared with CBA/J × BALB/c mouse matings [42] and in patients with RSA [12], implying a link between STMN1 and p53/TTP signals.

In conclusion, we identified TTP as a downstream target of p53 and as a mediator of the anti-proliferative effects of STMN1 in trophoblasts. Specifically, excessive p53 reduced STMN1 expression by upregulating TTP to suppress trophoblast migration and invasion at the early stage of placentation. Regrettably, because RSA samples are difficult to collect, number of samples is not enough. Further we will continue to collect more RSA samples to validate our discovery. Although, the relation between p53, STMN1 and TTP is complicated, many other elements that could affect them, including mechanism of TTP increasing. But our study provides an important foundation for future studies investigating mRNA destabilizing factors at the maternal–fetal interface. Trophoblast proliferation, migration, invasion, and endocrine secretion play important roles in a successful pregnancy. Along the way, we will also learn much about possible therapeutic strategies for preventing and/or treating pregnancy complications.
